# A Potential Therapeutic Strategy for Malignant Mesothelioma with Gene Medicine

**DOI:** 10.1155/2013/572609

**Published:** 2013-01-16

**Authors:** Yuji Tada, Hideaki Shimada, Kenzo Hiroshima, Masatoshi Tagawa

**Affiliations:** ^1^Department of Respirology, Graduate School of Medicine, Chiba University, 1-8-1 Inohana, Chuo-ku, Chiba 260-8670, Japan; ^2^Department of Surgery, School of Medicine, Toho University, 6-11-1 Omori-nishi, Ota-ku, Tokyo 143-8540, Japan; ^3^Department of Pathology, Tokyo Women's Medical University Yachiyo Medical Center, 477-96 Owada-Shinden, Yachiyo 276-8524, Japan; ^4^Division of Pathology and Cell Therapy, Chiba Cancer Center Research Institute, 666-2 Nitona, Chuo-ku, Chiba 260-8717, Japan; ^5^Department of Molecular Biology and Oncology, Graduate School of Medicine, Chiba University, 1-8-1 Inohana, Chuo-ku, Chiba 260-8670, Japan

## Abstract

Malignant mesothelioma, closely linked with occupational asbestos exposure, is relatively rare in the frequency, but the patient numbers are going to increase in the next few decades all over the world. The current treatment modalities are not effective in terms of the overall survival and the quality of life. Mesothelioma mainly develops in the thoracic cavity and infrequently metastasizes to extrapleural organs. A local treatment can thereby be beneficial to the patients, and gene therapy with an intrapleural administration of vectors is one of the potential therapeutics. Preclinical studies demonstrated the efficacy of gene medicine for mesothelioma, and clinical trials with adenovirus vectors showed the safety of an intrapleural injection and a possible involvement of antitumor immune responses. Nevertheless, low transduction efficiency remains the main hurdle that hinders further clinical applications. Moreover, rapid generation of antivector antibody also inhibits transgene expressions. In this paper, we review the current status of preclinical and clinical gene therapy for malignant mesothelioma and discuss potential clinical directions of gene medicine in terms of a combinatory use with anticancer agents and with immunotherapy.

## 1. Introduction of Mesothelioma

Malignant mesothelioma is a locally aggressive tumor of pleura or peritoneum and is refractory to conventional treatments. Mesothelioma is associated with occupational asbestos exposure in most of the cases, and a widespread asbestos usage generated a social concern in industrialized countries [1]. Moreover, many economical emerging countries have not yet legally inhibited an asbestos usage, which predicts a great number of the patients suffered in future. The latent period of mesothelioma is in general beyond 20 years, and no preventive method after asbestos exposure is currently available. Diagnosis at an early stage of the disease is often difficult because of nonspecific signs and symptoms, and consequently a large majority of the patients are found in an advanced stage. Interestingly, there is also an arousing concern about a widespread usage of nanosized particles for medical and industrial purposes, which may predispose to mesothelioma developments.

 Mesothelioma extends into organs in the vicinity and disturbs functions of vital organs, but infrequently metastasizes to distant organs until it develops into a terminal stage [2]. Invasion into vertebra or thoracic wall causes not only bone and neuropathic pain but compression of heart and great vessels, which results in cardiac tamponade, superior vena cava syndrome, and massive pleural effusion. Mesothelioma frequently penetrates into lung parenchyma and induces progressive respiratory failure. Suppressed local tumor growth within the pleural cavity alleviates their symptoms and is beneficial to the patients.

## 2. Current Standard Treatment for Mesothelioma

A standard treatment for mesothelioma consists of surgical resection, irradiation, and systemic chemotherapy. A small number of the patients with localized tumors can be subjected to surgery, either extrapleural pneumonectomy or pleurectomy with decortication. The former procedure is to remove pleura and lung en bloc, and the latter is to eliminate the involved pleura and to free the underlying lung to expand in the pleural cavity. A majority of the patients who undergo such radical debulking surgery, however, have a frequent local recurrence, and no further curative treatments are available in the recurrent cases. Mesothelioma in general is not insensitive to radiation, but the overall efficacy of radiation therapy is disappointing since irradiation to a widespread tumor area with a high radiation dose causes severe adverse effects, such as severe pneumonitis, myocarditis, and myelopathy due to spinal cord toxicity. Radiation therapy is therefore applicable only for a palliative purpose or in combination with surgery.

Mesothelioma is essentially resistant to cytotoxic chemotherapy. Any regimens including cisplatin, carboplatin, docetaxel, vinorelbine, and gemcitabine could not have prolonged the overall survival [3]. In contrast, a multitargeted antifolate agent, pemetrexed, in combination with cisplatin, has achieved the median survival time longer than chemotherapy with cisplatin alone (9.3 months versus 12.1 month) [4]. Currently, a combination of cisplatin and pemetrexed is the first-line standard regimen, and a combination of carboplatin plus either pemetrexed or raltitrexed also achieved similar clinical responses [5, 6]. There is, however, no reliable second-line chemotherapy regimen available at present [7]. A multi-modality therapy, consisting of induction chemotherapy, extrapleural pneumonectomy, and adjuvant irradiation, can produce a beneficial outcome to the patients, but it can be applicable to those only at a very early stage with a good performance status [8].

Molecular target therapy has been examined in mesothelioma patients. Mesothelioma expresses the epidermal growth factor receptor (EGFR) at a high level but the tyrosine kinase inhibitor, erlotinib, did not show any survival benefits in a phase II clinical trial [9]. Mesothelioma secretes angiogenic factors, platelet-derived growth factor and vascular endothelial growth factor (VEGF), both of which are also associated with cell proliferation and pleural effusion. Inhibition of angiogenesis produced antitumor responses and decreased pleural effusion [10], and therefore targeting angiogenesis was clinically examined for the therapeutic efficacy. A phase II study, however, demonstrated that antibody for VEGF, bevacizumab, with chemotherapy failed to prolong the progression free survival compared with the chemotherapy alone [11]. Taking these results together, molecular target medicine, as a single agent, could produce only a limited success, and no survival benefits have been observed even in a combination with other anticancer agents. Consequently, a majority of the patients are subject to take a best supportive care when the first-line chemotherapy becomes ineffective. A novel therapeutic strategy is definitely required to improve the survival and quality of life for mesothelioma patients.

## 3. Gene Therapy Targeting Genetic and Signal Pathways

Genetic analyses showed that mesothelioma has 3 characteristic genetic defects, and these deficits can be a target for the treatments. About 70–80% of mesothelioma specimens possess deficiency in the INK4A/ARF locus containing *p16* ^*INK4A*^ and *p14* ^*ARF*^ genes [12], about 40% in *neurofibromatosis type 2* gene (*NF2*) [13] and about 10% in *BRCA1 associated protein-1 *(*BAP1*) gene [14]. The *NF2 * gene-encoded protein, merlin, regulates several signal cascades, and the defect constitutively activates Yes-associated protein transcriptional coactivator. Transduction of the *NF2* gene in mesothelioma has not been examined probably due to the complexity of the downstream pathways. Deficient BAP1 is not specific to mesothelioma and is rather more often linked with other cancer types, and the biological significance is currently unknown. In contract, the downstream pathways of *p14 * and *p16 * genes were well characterized, and the genes are a good objective for mesothelioma treatments because the majority of mesothelioma has the wild-type *p53 * gene [12].

A genetic defect in the INK4A/ARF locus leads to the loss of the functions of both p53 and pRb tumor suppressors. A deficient p14 state augments MDM2 activity which facilitates p53 degradation through ubiquitin-proteasome pathways, and consequently generates a functional loss of p53 actions. Lack of p16 leads to the enhanced activities of cyclin-dependent kinase 4 and 6 and induces the phosphorylation of pRb. Loss of p21 induction due to the p53 deficiency does not suppress cyclin-dependent kinase 2 activities and favors pRb phosphorylation as well, which promotes cell cycle progression. Re-introduction of the defective *p14 *gene into *p14/p16*-defective mesothelioma cells thereby restored the sensitivity to p53-induced apoptosis and increased the dephosphorylation of pRb through p53-induced p21 actions [15]. Likewise, transduction of mesothelioma cells with the *p16* gene decreased pRb phosphorylation levels and inhibited cell cycle progression [16]. In fact, adenoviruses (Ads) expressing the *p14* gene (Ad-*p14*) and Ad-p16 induced the cell death of infected mesothelioma cells [15, 16]. Interestingly, Ad-16 produced greater antitumor effects than Ad-p14 [17, 18], and a combinatory use of Ad-p14 and Ad-p16 did not achieve better therapeutic effects than monotherapy with individual Ad [18]. Restoration of the p53-mediated pathways by these Ad can be effective only in mesothelioma cells with functional p53 downstream pathways. In contrast, the transduction of mesothelioma cells with Ad-p53 can also target even *p53*-mutated mesothelioma as well. 

Recently, Li et al. analyzed the effects of Ad-p53 transduction in *p14*/*p16*-defective mesothelioma [19], which is a direct way to reactivate the p53 pathways. Transduction with Ad-53 induced phosphorylation of p53 at serine 15 and 46 residues, a marker of the p53 activation, and augmented extrinsic apoptotic pathways through upregulating the death receptors. Induction of p21 further downregulated hyperphosphorylated pRb and inhibited cell cycle progression. Ad-p53 transduction not only produced apoptotic tumor cell death but synergistic cytotoxicity with the first-line anticancer agents, cisplatin, and pemetrexed. Moreover, Ad-p53-mediated antitumor effects and the combinatory use with cisplatin were demonstrated in an orthotopic animal model. These data collectively indicate that the reactivation of the defective p53 pathways is a promising direction of gene therapy for mesothelioma.

Biochemical analyses showed that 3 major signaling pathways were activated in mesothelioma, EGF and EGFR, VEGF and the receptors VEGFR1 and VEGFR2, and hepatocyte growth factor (HGF) and the receptor c-Met. As mentioned above, clinical studies demonstrated that the tyrosine kinase inhibitor for EGFR and antibody for VEGFR failed to prolong the patient survivals [9, 11]. An inhibitor for the c-Met kinase is now under investigation for the clinical efficacy. Preclinical studies to inhibit the HGF/c-Met pathways with gene medicine were conducted for mesothelioma. The HGF/c-Met pathways have a crucial role in the invasion of tumors and in metastasis, and previous studies in fact demonstrated that the inhibition of the pathway suppressed tumor infiltration into neighboring tissues [20]. NK4, a secretary N-terminal fragment of HGF *α*-chain, binds to c-Met receptors as a competitive antagonist and subsequently blocks the HGF/c-Met pathways. Suzuki et al. demonstrated that nonreplicating Ad expressing the *NK4* gene (Ad-NK4) produced antitumor effects against mesothelioma [21]. Interestingly, Ad-NK4 also inhibits the VEGF-mediated angiogenesis although the mechanism is uncharacterized. Recently, Sakai et al. suggested the mechanism that NK4 did not directly block the interactions between VEGF and the receptors, but rather indirectly suppressed the VEGF-mediated angiogenic environments through impairing fibronectin assembly [22]. A clinical study to examine the safety and the efficacy of Ad-NK4 is currently planned for mesothelioma patients.

## 4. Gene Therapy with Oncolytic Viruses

Replication of viruses can induce host cell death and the viral proliferation with tumorspecificity is a key for oncolytic viruses. There are several strategies to produce such viruses depending on the replication mechanisms of respective viruses. Ads are frequently used as oncolytic viruses among replication-competent viruses. The typical type of oncolytic Ad is to activate the viral E1A, a transcriptional factor to drive cell cycle progression and to activate viral genes necessary for viral replication. A transcriptional regulatory region of a gene whose expression is elevated in target tumors but not in normal cells is often used for a tumor-specific E1A activation. Another type is defective viruses that lost functions to activate cellular proteins such as a tumor suppressor. Practically, replication of oncolytic Ad and subsequent cell death are not precisely specific to target tumors in most of the cases since differential transcriptional propensities between tumorous and nontumorous cells are unclear and often transitional. For example, a transcriptional activity of the so-called putative tumor-specific promoters that are used to activate the *E1A* gene is often proportionately linked with the proliferation ability of infected cells or is closely associated with tissue specificity of the target cells. Tumor specificity at a transcriptional level is thus often irrelevant to the distinctive difference between tumors and normal tissues. A defect in virus inactivation systems in type-1 interferon (IFN) pathways, which is regarded as a mechanism of tumor-specific viral replication, is not necessary to be a major differential property between them. Aiming at such differentials in fact did not induce tumor-specific cytotoxicity in a strict sense, and we often observed the nonspecific destroy of neighboring nontumorous cells as well. Nevertheless, the Ad-mediated cytotoxicity was always greater in tumors than in normal cells, and such preferential killing of tumors was practically sufficient in terms of antitumor responses. The augmented cell death in tumors is due to differential cell proliferation between tumors and normal tissues, and Ad replication is enhanced in rapidly proliferating cells rather than in static cells. The most important merit of oncolytic viruses is to destroy tumors with keeping host immunity intact, which stands in a sharp contrast with conventional chemotherapy and radiotherapy that are often suppressive to defense mechanisms as discussed later.

Gene therapy with oncolytic viruses has not yet been clinically tested for mesothelioma patients but several types of oncolytic viruses such as Ad [23], retrovirus [24], herpes simplex virus (HSV) [25], and measles virus [26] were examined for the therapeutic effects in preclinical settings. The mechanisms of tumor-specific cell lysis are different among the vectors and the cytotoxic activities were not even specific to mesothelioma. These viruses were also cytocidal to other cancer types, and it is difficult to determine at this moment what types of viruses are suitable for mesothelioma treatments. The optimal virus types will be determined by clinical trials in future.

The first oncolytic viruses commercially available in the world are Ad defective of E1B-55 kD molecules. Chinese state food and drug administration approved the Ad for head and neck cancer treatments in 2005. A typical role of E1B-55 kD molecules is to bind to p53 protein and to inactivate the p53 pathways. Ad infection promptly induces E1A, one of the immediate early gene products, and E1A subsequently induces p53 expression in the host cells. E1B-55 kD-defective Ads thereby augment p53 expression levels in infected mesothelioma cells since E1A-induce p53 expression is not inhibited by E1B-55 kD molecules. Moreover, augmented p53 induces p21, and pRb is subsequently dephosphorylated as found in Ad-p53-infected mesothelioma. Yamanaka et al. demonstrated that the E1B-55 kD-defective Ad induced G0/G1 phase arrest and then apoptosis with cleavages of caspases in mesothelioma cells bearing the wild-type *p53 *gene [27]. In addition, the E1B-55 kD-defective Ad produced synergistic combinatory cytotoxicity with cisplatin and pemetrexed, and intrapleural injection of the Ad suppressed mesothelioma growth *in vivo* in an orthotopic animal model. The defective Ads, thus, have two mechanisms to achieve antitumor effects through augmenting the p53 pathways and viral replication.

## 5. Clinical Trials for Mesothelioma with Replication-Incompetent Ad

Gene therapy has several advantages in mesothelioma treatments because mesothelioma develops in a closed cavity and is localized within the cavity until it proceeds to a terminal stage. Suppression of local tumor growth is thereby beneficial to the patients and an intrapleural administration of vectors is technically feasible. Injected vectors will be localized within the cavity without being rapidly washed out or diluted by bloodstream, and a constant respiratory lung movement facilitates vector distribution within the pleural cavity. These conditions are favorable to increase the transduction efficacy. There have been 5 major clinical trials reported, which included 3 phase I and 2 pilot studies, using nonreplicating Ad (3 phase I and 1 pilot studies) and vaccinia viruses (1 pilot study) (Table 1) [28]. The genes used in these clinical trials were HSV-thymidine kinase (HSV-TK) (as suicide gene therapy) [29], interleukin-2 [30], and interferon-*α* and -*β* (IFN-*α* and -*β*) [31–33]. All the patients except those who received the *interleukin-2* gene in a vaccine vector (via an intratumoral administration) were treated with an intrapleural injection of Ad. About 60 patients were included in the Ad-injected cases, and all of them showed no severe adverse events, demonstrating that an intrapleural injection of Ad was conducted safely. These studies showed the transgene expression in mesothelioma but also revealed that the viral spread was limited only around the injection sites or in a surface region even with good amounts of Ad used. Consequently, the overall clinical responses were not significant. These studies, however, reported a few patients that showed a good clinical outcome several months after the Ad administrations. The delayed responses with the low transduction efficacy suggested that these beneficial cases were due to possible immune responses even though some of them were treated with the suicide gene therapy which aimed at direct cell death. The studies showed that some of the patients developed antibody that reacted with several unidentified molecules found in human mesothelioma cell lines. Interestingly, the antibody was not detected in the pretreated serum and the molecule species detected by the antibody were different among the patients. The properties of these molecules remained uncharacterized, but they might be putative tumor antigen(s) that were commonly shared among mesothelioma. These clinical data collectively suggested that immune responses were generated against mesothelioma, but it was unclear whether the antibody played a certain role in the antitumor effects. Neutralizing antibody against Ad was also produced by an intrapleural administration with a similar kinetics as found in the cases of a systemic administration. The kinetics data suggested that Ads were readily transferred to regional lymph nodes along the thorax and/or were directly absorbed into bloodstream via mesothelium. A clinical study demonstrated that Ads injected in the intrapleural cavity were detected in serum for a week, but the administration did not induce any severe liver damages, a typical adverse reaction caused by the systemic administration of type 5 Ad [31]. Our preliminary data suggested that lung tissues became positive for Ad vectors when they were administered in the pleural cavity. These data suggest that Ads in the pleural cavity have two possible biodistribution routes, lymph nodes and bloodstream. Generation of antivector antibody can suppress the hematogenous transfer of Ad into liver, but it is uncharacterized as to how much of the antibody titers were required to inhibit the hepatic integration of Ad.

## 6. Optimizing Viruses-Mediated Gene Therapy

The major disadvantage of gene therapy, with either replication-incompetent or oncolytic viruses, is inadequate transduction efficacy due to low-leveled infectivity and an insufficient viral spread within tumor tissues. Production of antiviral neutralizing antibody was inhibitory to the transduction especially when viruses were repeatedly administered. Shortening an interval period of repeated Ad injections and administrating an immunosuppressive agent are possible options to reduce antibody production although further investigations are required to examine clinical feasibility of these options. Recent clinical studies for mesothelioma showed that an intrapleural injection of Ad at a 3-days interval was favorable to decrease subsequent antibody upsurge in comparison with that at a 7-day interval and demonstrated that the short interval increased the transgene expression mediated by the second Ad injection [32, 33]. Willmon et al. reported that cyclophosphamide administration increased replication of oncolytic vesicular stomatitis viruses coinjected, but did not enhance the viruses-mediated cytotoxicity [34], indicating that an anticancer agent at a low dose suppressed antibody production and viral replication levels were irrelevant to the antitumor activities. Saito et al. reported a carrier cells-based system, so-called a Trojan horse delivery system [35]. Administration of irradiated tumor cells that were preinfected with oncolytic Ad evaded neutralizing antibody-mediated inactivation of Ad and produced better therapeutic effects than direct intratumoral Ad injections at the same virus amounts. Administration of oncolytic viruses together with recombinant IFN-*γ* or that of oncolytic viruses engineered to express the *IFN-*γ** gene effectively inhibited antibody production against the viruses since IFN-*γ* decreased humoral immunity [36]. There are several ways to deal with the generation of antivector antibody but further investigations are required to optimize the methodology.

Viral infectivity depends on an expression level of the receptors that mediate the viral entry into target cells. The main cellular receptor of type 5 Ad is coxsackie and adenovirus receptor (CAR), and the expression level is often downregulated in a number of human tumors including mesothelioma. Tumors expressing CAR at a low level are thereby resistant to type 5 Ad-mediated gene transduction. A recent study showed that CD46 expression was not downregulated on mesothelioma cells [37], and recombinant type 5 Ad which replaced the fiber-knob region with that of type 35 Ad, shifting the Ad receptors from CAR to CD46 molecules, dramatically improved the infectivity [38]. Furthermore such a switching receptor-binding site may hinder the rapid production of neutralizing antibody since humans are less frequently exposed to type 35 Ad and many persons have not yet primed for the type 35 viral antigens. Chemical agents may augment the viral effects; for example, a calcium blocker, verapamil, and enhanced efficacy of oncolytic Ad administered simultaneously [39, 40]. An exact mechanism about the verapamil-mediated enhancement of antitumor effects remains unclear, but the inhibition of calcium influx may augment Ad releases and improve the spread into the vicinity.

Mesothelioma produces a large amount of the latent form of transforming growth factor-*β* (TGF-*β*) which stimulates fibrous stroma formation as well as immune tolerance that protects the tumors from host defense mechanisms. Seth et al. developed oncolytic Ad armed with the soluble TGF-*β* receptor II fused with the Fc receptors [41]. The fusion protein bound to TGF-*β* as a decoy and inhibited TGF-*β*-dependent transcription in tumors, which subsequently augmented viral replications in the infected tumor cells. Moreover, the decoy inhibited collagen formation around the tumors. Extracellular matrixes in tumor nests contribute to resistance to various therapies by preventing the penetration of therapeutic agents including viral vectors [42]. Spread of the viruses is strongly impaired by stroma cells and matrixes within tumor nests. Watanabe et al. demonstrated that coexpressed heparanase and endoglucuronidase which degraded heparin sulfate decreased a physiological barrier within tumors and increased the viral permeability [43]. Cheng et al. further demonstrated that the intratumoral administration of Ad expressing the *matrix metalloproteinase-8* gene together with oncolytic Ad improved the viral spread and enhanced the oncolytic activity [44]. Relaxin, a peptide hormone related to insulin or insulin-like growth factor, is also a candidate since it inhibits collagen formation by fibroblasts and breaks down the matrixes by increasing matrix metalloproteinase expression [45, 46]. On the other hand, overexpression of relaxin and other matrix-degrading enzymes arouse concern about the possible increase of metastasis by enhancing the release of tumor cells from the primary site.

## 7. Possible Strategies to Augment the Efficacy of Virotherapy

Mesothelioma is nonimmunogenic tumors in most of the cases and can evade antitumor immunity. Triggering local inflammation in the vicinity of mesothelioma may break this tolerance. A virus vector is one of the good platforms for immunotherapy because viruses are immunogenic and cause inflammatory and immune responses in contrast to conventional chemotherapy and radiotherapy. Destruction of tumor cells with viral-mediated gene transduction or with oncolytic viruses will facilitate the release of putative tumor antigens, and local inflammation triggers subsequent cell-mediated antitumor immunity by enhancing the maturation of dendritic cells that engulf the released tumor-associated antigens. Arming viruses with immune stimulatory molecules can further improve the antitumor efficacy. For instance, coexpression of IFN-*β* or IFN-*γ* in the context of oncolytic viruses dramatically enhanced gene transfer and the oncolytic actions despite the antiviral responses since IFNs augment host immune responses and moreover induce antiangiogenesis [26, 36, 47]. Oncolytic Ad expressing the *CD40 ligand *gene enhanced antitumor immunity by upregulating the production of T-helper type 1 cytokines such as IFN-*γ*, tumor necrosis factor-*α*, and chemokine (C-C motif) ligand 5, all of which maturate dendritic cells to present tumor antigens efficiently [23].

Proteins preferentially expressed in tumors have many values for cancer therapy. A regulatory region of the encoding gene can be used to activate a transgene or viral genes in the case of oncolytic viruses. When the expression is quantitatively and qualitatively specific to mesothelioma, the molecules can be a target of immunotherapy and a biomarker for diagnostics. Survivin is an antiapoptotic protein that inhibits caspase activity, and the expression is tightly related cell growth. The expression is upregulated in a variety of cancer including mesothelioma. Oncolytic Ad with the survivin promoter thereby produced tumor-specific cell lysis with minimal adverse effects to normal cells [48, 49]. Moreover, a survivin peptide is being tested as a possible therapeutic vaccine that evokes antitumor immune responses. Midkine is a basic heparin-binding growth factor with a retinoic acid-responsive action. It has multiple biological activities such as cell proliferation, angiogenesis, and antiapoptosis, all of which are closely associated with cancer developments [50]. Kubo et al. showed that replication-competent Ad driven by the midkine promoter achieved antitumor responses against mesothelioma [51]. Recently studies on proteomics revealed candidates of protein markers useful for diagnosis of mesothelioma [52]. For example, calretinin, a calcium-binding protein, was frequently expressed in any types of mesothelioma [53]. Serum mesothelin levels were elevated in patients of mesothelioma, and the levels were influenced by the efficacy of treatments [54]. Mesothelin is also a target tumor antigen for immunotherapy, and antimesothelin antibody is being tested for its antibody-mediated cytotoxicity. These markers are not completely specific to mesothelioma but are useful to distinguish mesothelioma from lung adenocarcinoma since both tumors are clinically and sometimes pathologically difficult to settle differential diagnosis.

Oncolytic viruses enforced with apoptosis induction or with the inhibition of oncogene products are currently tested. For example, oncolytic Ad expressing tumor necrosis factor alpha-related apoptosis-inducing ligand achieved a significant cytotoxicity to lung carcinoma [55]. Zhang et al. developed oncolytic Ad fused with small interfering RNA for the *K-ras *gene to treat various kinds of human cancers [56]. Oncolysis with a gene silencing system, however, needs further investigations as to whether knocking down the target molecules inhibits the viral proliferation.

Blocking angiogenesis for feeding vessels is one of the major strategies for cancer treatments. He et al. developed the E1B-55 kD-defective Ad expressing the *canstatin* gene which encoded an antiangiogenic protein and demonstrated the antitumor effects on pancreatic carcinoma [57]. Ad-NK4 is one of the angiogenesis-target therapies, and a combinatory use of oncolytic viruses and expressed NK4 would be of interest for mesothelioma treatments.

## 8. Conclusions

It is almost 10 years since the Ad-p53 was approved in China as the first gene medicine worldwide for head and neck cancer, and the E1B-55 kD-defective Ad has 7-year clinical experiences in major Chinese hospitals. Notwithstanding, gene therapy has not received wide recognition as a novel therapeutics for any types of cancer including mesothelioma. It is probably due to some difficulties to collect enough evidences regarding the therapeutic efficacy in comparison with other anticancer drugs. In this context, gene therapy does not have to be used as a monotherapy but rather should be combined with other conventional modalities to seek for the so-called add-on effects since gene medicine itself is demonstrated as safe in many clinical trials. Viruses-based gene medicine can be useful from many clinical viewpoints such as an agent for induction therapy, and even as an adjuvant for a palliative treatment owing to the efficient immune-stimulatory activity. The mechanism of viruses-mediated cell death is not yet completely understood although some studies suggested a possible involvement of autophagy. An important point is that the cell death system is different from those of other therapeutics, and the unique mechanism can widen its potential utility as cancer treatments. Currently gene therapy and virotherapy are still one of the treatment strategies under investigations, but accumulating clinical data suggest that it can produce antitumor effects which have not been achieved by other therapies [58]. Mesothelioma is obviously one of the target tumors for gene therapy, and in fact several clinical studies with oncolytic viruses are now in progress. Gene therapy currently remains an experimental approach for mesothelioma treatments, but the preceding clinical trials provided many points to be considered for the future application of gene therapy. Gene therapy in combination with other modalities will be surely an alternative for current cancer therapeutics, but biomarkers to predict the efficacy need to be developed to determine the clinical eligibility.

## Figures and Tables

**Table 1:** Clinical gene therapy trials for mesothelioma.

Phase study	Vector	Expressed gene	Patient number	Virus titer used [reference]
Pilot	Vaccinia	Interleukin-2	6	Not reported [30]^1^
Pilot	Ad	IFN-*α*2b	9	3 × 10^11^–1 × 10^12^ vp [33]^2^
I	Ad	HSV-TK	13 21	5 × 10^10^–5 × 10^12^ vp 1.5 × 10^13^–5 × 10^13^ vp [29]^3^
I	Ad	IFN-*β*	8	9 × 10^11^–3 × 10^12^ vp [31]^4^
I	Ad	IFN-*β*	10	3 × 10^11^–3 × 10^12^ vp [32]^5^

^1^Intratumoral injection. The transgene expression was observed despite antivaccinia antibody generated.

^2^Twice intrapleural injections of E1/E3-deleted Ad at 3-day interval. One case showed more than 50% tumor reduction which was judged with a radiographic assessment on day 64. vp: virus particle.

^3^A single intrapleural injection of either E1/E3-deleted or E1/E4-deleted Ad. Two long-term survivors (more than 6.5 years) were included in the E1/E4-deleted Ad-injected group.

^4^A single intrapleural injection of E1/E3-deleted Ad. Maximum tolerance dose was judged as 9 × 10^11^ vp. Polymerase chain reaction detected the viral shedding in serum up to day 4 and in pleural fluid up to day 42.

^5^Twice intrapleural injections of E1/E3-deleted Ad at 7-day interval. No maximum tolerance dose was reached. Neutralizing antibody was generated until day 7.

## Abbreviations

EGFR: Epidermal growth factor receptor
VEGF: Vascular endothelial growth factor
Ads: Adenoviruses
HGF: Hepatocyte growth factor
IFN: Interferon
HSV: Herpes simplex virus
HSV-TK: Herpes simplex virus-thymidine kinase
CAR: Coxsackie and adenovirus receptor
TGF-*β*: Transforming growth factor-*β*
vp: Viral particles.
